# Exact Expressions for Kullback–Leibler Divergence for Univariate Distributions

**DOI:** 10.3390/e26110959

**Published:** 2024-11-07

**Authors:** Victor Nawa, Saralees Nadarajah

**Affiliations:** 1 Department of Mathematics and Statistics, University of Zambia, Lusaka 10101, Zambia; vnawa@yahoo.com; 2 Department of Mathematics, University of Manchester, Manchester M13 9PL, UK

**Keywords:** Bessel function, beta function, confluent hypergeometric function, gamma function, Gauss hypergeometric function

## Abstract

The Kullback–Leibler divergence (KL divergence) is a statistical measure that quantifies the difference between two probability distributions. Specifically, it assesses the amount of information that is lost when one distribution is used to approximate another. This concept is crucial in various fields, including information theory, statistics, and machine learning, as it helps in understanding how well a model represents the underlying data. In a recent study by Nawa and Nadarajah, a comprehensive collection of exact expressions for the Kullback–Leibler divergence was derived for both multivariate and matrix-variate distributions. This work is significant as it expands on our existing knowledge of KL divergence by providing precise formulations for over sixty univariate distributions. The authors also ensured the accuracy of these expressions through numerical checks, which adds a layer of validation to their findings. The derived expressions incorporate various special functions, highlighting the mathematical complexity and richness of the topic. This research contributes to a deeper understanding of KL divergence and its applications in statistical analysis and modeling.

## 1. Introduction

Kullback–Leibler divergence (KLD) is a fundamental concept in information theory that serves as a quantitative measure of the difference between two probability distributions. Specifically, it assesses how one probability distribution diverges from a second, designated as the reference distribution. This measure is particularly valuable in various fields, including machine learning, statistics, and data science, where it is employed to evaluate the effectiveness of a model’s predicted probability distribution in approximating the actual distribution of observed data.

One of the key characteristics of the KLD is its asymmetry; that is, the divergence calculated from distribution A to distribution B is not necessarily equal to the divergence from B to A. This property distinguishes the KLD from other distance metrics, such as the Euclidean distance, that are symmetric in nature.

The KLD finds extensive applications in optimizing machine learning models. By minimizing the KLD between the predicted probability distribution generated by a model and the true underlying distribution of the data, practitioners can enhance the accuracy and performance of their models. This optimization process is crucial for developing models that not only fit the training data well but also generalize effectively to unseen data.

Suppose *X* is a continuous random variable with one of two probability density functions: $f_{i}\left(\cdot ;\theta_{i}\right)$, i=1,2 parameterized by $\theta_{i}$, i=1,2. The KLD between $f_{1}\left(\cdot ;\theta_{1}\right)$ and $f_{2}\left(\cdot ;\theta_{2}\right)$ (Kullback and Leibler [1]) is defined by

(1) $$\begin{array}{c} KLD\left(f_{1}\left(x\right),f_{2}\left(x\right)\right)=E\left[\log\frac{f_{1}\left(X;\theta_{1}\right)}{f_{2}\left(X;\theta_{2}\right)}\right], \end{array}$$

where the expectation is with respect to $f_{1}\left(\cdot ;\theta_{1}\right)$.

Comprehensive accounts of the theory and applications of (1) can be found in [2,3,4,5,6]. Various applications of (1) are discussed in these books. Ref. [2] discussed applications to contingency tables, linear hypotheses, multivariate analyses and linear discriminant functions. Ref. [5] discussed applications to goodness-of-fit tests, testing log–linear models, contingency tables and testing in general populations. Ref. [6] discussed measures of robustness, hypothesis testing, techniques for inlier modification, weighted likelihood estimation, multinomial goodness-of-fit testing and applications in engineering.

Because of the increasing use of the KLD, it is useful to have exact expressions for (1). We are not aware of any existing comprehensive collection of exact expressions for (1). A comprehensive collection of exact expressions for (1) for multivariate and matrix-variate distributions was provided only recently by Nawa and Nadarajah [7]. The aim of this paper is to derive exact expressions for (1) for over sixty univariate distributions. The exact expressions are given in Section 2. A sketch of derivations is given in Section 5, but full derivations can be obtained from the corresponding author. A numerical check for the correctness of the expressions in Section 2 is performed in Section 6. Finally, conclusions and future work are noted in Section 7. The distributions considered in this paper are continuous. We shall not be considering discrete distributions including mixtures.

The expressions in Section 2 involve various special functions, including the gamma function defined by

$$\begin{array}{c} \Gamma \left(a\right)=\int_{0}^{\infty }t^{a-1}\exp\left(-t\right)dt; \end{array}$$

the incomplete gamma function defined by

$$\begin{array}{c} \Gamma \left(a,x\right)=\int_{x}^{\infty }t^{a-1}\exp\left(-t\right)dt; \end{array}$$

the digamma function defined by

$$\begin{array}{c} \psi \left(a\right)=\frac{d\log\Gamma (a)}{da}; \end{array}$$

the beta function defined by

$$\begin{array}{c} B\left(a,b\right)=\int_{0}^{1}t^{a-1}{\left(1-t\right)}^{b-1}dt; \end{array}$$

the incomplete beta function defined by

$$\begin{array}{c} B_{x}\left(a,b\right)=\int_{0}^{x}t^{a-1}{\left(1-t\right)}^{b-1}dt; \end{array}$$

the standard normal probability density function defined by

$$\begin{array}{c} \phi \left(x\right)=\frac{1}{\sqrt{2\pi }}\exp\left(-\frac{x^{2}}{2}\right); \end{array}$$

the standard normal cumulative distribution function defined by

$$\begin{array}{c} \Phi \left(x\right)=\frac{1}{\sqrt{2\pi }}\int_{-\infty }^{x}\exp\left(-\frac{t^{2}}{2}\right)dt; \end{array}$$

the modified Bessel function of the second kind defined by

$$\begin{array}{c} K_{\nu }\left(x\right)=\left\{\begin{array}{cc} \frac{\pi \csc(\pi \nu )}{2}\left[I_{-\nu }\left(x\right)-I_{\nu }\left(x\right)\right], & \mathrm{if}\nu \notin Z,\\ \lim_{\mu \to \nu }K_{\mu }\left(x\right), & \mathrm{if}\nu \in Z; \end{array}\right. \end{array}$$

the modified Bessel function of the first kind of order ν defined by

$$\begin{array}{c} I_{\nu }\left(x\right)=\sum_{k=0}^{\infty }\frac{1}{\Gamma (k+\nu +1)k!}{\left(\frac{x}{2}\right)}^{2k+\nu }; \end{array}$$

the confluent hypergeometric function of one variable defined by

F11a;b;x=∑k=0∞(a)k(b)kxkk!;

the Gauss hypergeometric function of one variable defined by

F12a,b;c;x=∑k=0∞(a)k(b)k(c)kxkk!;

the degenerate hypergeometric series of two variables defined by

$$\begin{array}{c} \Phi_{1}\left(a,b,c;x,y\right)=\sum_{m=0}^{\infty }\sum_{n=0}^{\infty }\frac{{\left(a\right)}_{m+n}{\left(b\right)}_{n}x^{m}y^{n}}{{\left(c\right)}_{m+n}m!n!}; \end{array}$$

the degenerate hypergeometric function of two variables defined by

$$\begin{array}{c} F_{1}\left(a,b,c;d;x,y\right)=\sum_{m=0}^{\infty }\sum_{n=0}^{\infty }\frac{{\left(a\right)}_{m+n}{\left(b\right)}_{m}{\left(c\right)}_{n}x^{m}y^{n}}{{\left(d\right)}_{m+n}m!n!}, \end{array}$$

where ${\left(f\right)}_{k}=f\left(f+1\right)\cdots \left(f+k-1\right)$ denotes the ascending factorial. The properties of these special functions can be found in [8,9]. In-built routines for their computations are available in packages like Maple, Mathematica and Matlab. These packages allow for arbitrary precision, and so many of the expressions for the KLD in Section 2 can be computed easily with arbitrary precisions. Arbitrary precisions may not be achieved if one resorts to (1) for computing the KLD, as this will require numerical integration. Numerical integration may be prone to errors and may be less efficient than algorithms imbedded in the in-built routines. Moreover, the coding of (1) will take time. However, some of the expressions for the KLD in Section 2 involve infinite sums. The KLD in these cases can be computed more efficiently by direct numerical integration.

## 2. Exact Expressions

Section 3 provides known expressions for (1) and references. Section 4 provides new expressions for (1).

## 3. Known Expressions

Known expressions for (1) for the confluent hypergeometric beta distribution [10], the exponential distribution, the gamma distribution, the generalized gamma distribution [11], the Gumbel distribution [12], the normal distribution, the Rayleigh distribution and the Weibull distribution [13] and the corresponding references are given in Table 1.

## 4. New Expressions

Table 2 provides new expressions for (1) for a generalized uniform distribution, the asymmetric Laplace distribution [22], the beta distribution, the beta exponential distribution [23], the beta prime distribution, the Burr distribution [24], the Cauchy distribution, the chi distribution, the chi-square distribution, the continuous Bernoulli distribution, the exponentiated exponential distribution [25], the *F* distribution, the Fréchet distribution [26], the gamma-Gompertz distribution, the generalized beta distribution of the first kind [27], the generalized beta distribution of the second kind [27], the generalized inverse Gaussian distribution, the generalized logistic distribution (types I-IV), the generalized Pareto distribution [28], the Gompertz distribution [29], the half-Cauchy distribution, the half-logistic distribution, the half-normal distribution, the half-Student’s *t*-distribution, the harmonic distribution, the inverse chi-square distribution, the inverse exponential distribution, the inverse gamma distribution, the inverse Gaussian distribution [30], the inverse Nakagami distribution, the inverse Rayleigh distribution, the *J*-shaped distribution [31], the Kumaraswamy distribution [32], the Laplace distribution, Libby and Novick [33]’s beta distribution, the Lindley distribution [34], the logit normal distribution, the Lomax distribution [35], the Nakagami distribution [36], the omega distribution [37], the Pareto type I distribution [38], the power function distribution of type I, the power function distribution of type II, the *q*-exponential distribution [39], the scaled inverse chi-square distribution, Schulz [40]’s distribution, Student’s *t*-distribution [41], the transmuted exponential distribution [42] and the truncated normal distribution.

## 5. A Sketch of the Derivations Given in Section 4

In this section, we provide sketches of the derivations of the expressions given in Table 2. For the generalized uniform distributions, using the fact that

$$\begin{array}{c} \int_{a}^{b}x^{\alpha }\log xdx={\left.\frac{d}{d\beta }\int_{a}^{b}x^{\alpha +\beta }dx\right|}_{\beta =0}={\left.\frac{d}{d\beta }\frac{b^{\alpha +\beta +1}-a^{\alpha +\beta +1}}{\alpha +\beta +1}\right|}_{\beta =0}, \end{array}$$

we obtain the result. For the asymmetric Laplace distributions [22], straightforward calculations prove the result. For the beta distributions, using the facts

$$\begin{array}{c} \int_{0}^{1}x^{\alpha }{\left(1-x\right)}^{\beta }\log xdx={\left.\frac{d}{d\gamma }\int_{0}^{1}x^{\alpha +\gamma }{\left(1-x\right)}^{\beta }dx\right|}_{\gamma =0}={\left.\frac{d}{d\gamma }B\left(\alpha +\gamma +1,\beta +1\right)\right|}_{\gamma =0} \end{array}$$

and

$$\begin{array}{c} \int_{0}^{1}x^{\alpha }{\left(1-x\right)}^{\beta }\log\left(1-x\right)dx={\left.\frac{d}{d\gamma }\int_{0}^{1}x^{\alpha }{\left(1-x\right)}^{\beta +\gamma }dx\right|}_{\gamma =0}={\left.\frac{d}{d\gamma }B\left(\alpha +1,\beta +\gamma +1\right)\right|}_{\gamma =0}, \end{array}$$

we obtain the result. For the beta exponential distributions [23], using the expansion

(2) $$\begin{array}{c} \log\left[1-\exp(-\alpha x)\right]=-\sum_{k=1}^{\infty }\frac{\exp(-k\alpha x)}{k} \end{array}$$,

along with known expressions for expectations and the moment-generating function of a beta exponential random variable (see [23]), we obtain the result. For the beta prime distributions, using the facts

$$\begin{array}{c} \int_{0}^{\infty }x^{\alpha -1}{\left(1+x\right)}^{-\alpha -\beta }\log xdx={\left.\frac{d}{d\gamma }\int_{0}^{\infty }x^{\alpha +\gamma -1}{\left(1+x\right)}^{-\alpha -\beta }dx\right|}_{\gamma =0}={\left.\frac{d}{d\gamma }B\left(\alpha +\gamma +1,\beta -\gamma \right)\right|}_{\gamma =0} \end{array}$$

and

$$\begin{array}{c} \int_{0}^{\infty }x^{\alpha -1}{\left(1+x\right)}^{-\alpha -\beta }\log\left(1+x\right)dx={\left.\frac{d}{d\gamma }\int_{0}^{1}x^{\alpha -1}{\left(1+x\right)}^{\gamma -\alpha -\beta }dx\right|}_{\gamma =0}={\left.\frac{d}{d\gamma }B\left(\alpha ,\beta -\gamma \right)\right|}_{\gamma =0}, \end{array}$$

we obtain the result. For Burr distributions [24], using the expansion

(3) $$\begin{array}{c} \log\left(1+z\right)=\sum_{k=1}^{\infty }\frac{{\left(-1\right)}^{k-1}z^{k}}{k}, \end{array}$$

(4) $$\begin{array}{c} \int_{0}^{\infty }\log xf\left(x\right)dx={\left.\frac{d}{d\alpha }\int_{0}^{\infty }x^{\alpha }f\left(x\right)dx\right|}_{\alpha =0}={\left.\frac{d}{d\alpha }E\left(X^{\alpha }\right)\right|}_{\alpha =0} \end{array}$$

and known expressions for expectations of a Burr random variable (see [24]), where *X* is a random variable with probability density function f(x), we obtain the result. For the Cauchy distributions, using the expansion given in (3) and known expressions for expectations of a Cauchy random variable, we obtain the result. For the chi distributions, calculations using (4) and expressions for expectations of a chi random variable prove the result. For the chi-square distributions, calculations using (4) and expressions for expectations of a chi-square random variable prove the result. For the continuous Bernoulli distributions, straightforward calculations using expectations of a continuous Bernoulli random variable prove the result.

For the exponentiated exponential distributions [25], using the expansion given in (2), along with known expressions for expectations and the moment-generating function of an exponentiated exponential random variable (see [25]), we obtain the result. For the *F* distributions, using the facts

$$\begin{array}{cc} \int_{0}^{\infty }x^{\alpha }{\left(1+ax\right)}^{-\beta }\log xdx\; & ={\left.\frac{d}{d\gamma }\int_{0}^{\infty }x^{\alpha +\gamma }{\left(1+ax\right)}^{-\beta }dx\right|}_{\gamma =0}\\ \; & ={\left.\frac{d}{d\gamma }\frac{B(\beta -\alpha -\gamma -1,\alpha +\gamma +1)}{a^{\alpha +\gamma +1}}\right|}_{\gamma =0} \end{array}$$

and

$$\begin{array}{cc} \int_{0}^{\infty }x^{\alpha }{\left(1+ax\right)}^{-\beta }\log\left(1+ax\right)dx\; & ={\left.\frac{d}{d\gamma }\int_{0}^{\infty }x^{\alpha }{\left(1+ax\right)}^{\gamma -\beta }dx\right|}_{\gamma =0}\\ \; & ={\left.\frac{d}{d\gamma }\frac{B(\beta -\alpha -\gamma -1,\alpha +1)}{a^{\alpha +1}}\right|}_{\gamma =0}, \end{array}$$

we obtain the result. For Fréchet distributions [26], calculations using (4) and expressions for expectations of a Fréchet random variable prove the result. For the gamma-Gompertz distributions, using the expansion given in (2), along with known expressions for expectations and the moment-generating function of a gamma-Gompertz random variable, we obtain the result. For the generalized beta distributions of the first kind [27], using the expansions given in (3) and (4) and known expressions for expectations of a generalized beta random variable of the first kind (see [27]), we obtain the result. For the generalized beta distributions of the second kind [27], using the expansions given in (3) and (4) and known expressions for expectations of a generalized beta random variable of the second kind (see [27]), we obtain the result. For the generalized inverse Gaussian distributions, using (4) and known expressions for expectations of a generalized inverse Gaussian variable, we obtain the result. For the generalized logistic distributions (type I), using

(5) $$\begin{array}{c} \int_{-\infty }^{\infty }\log\left[1+\exp(-x)\right]f\left(x\right)dx={\left.\frac{d}{d\alpha }\int_{-\infty }^{\infty }{\left[1+\exp(-x)\right]}^{\alpha }f\left(x\right)dx\right|}_{\alpha =0}, \end{array}$$

where f(x) denotes a probability density function, and setting $y={\left[1+\exp(-x)\right]}^{-1}$, we obtain the result. For the generalized logistic distributions (type II), using (5) and setting $y={\left[1+\exp(-x)\right]}^{-1}$, we obtain the result. For the generalized logistic distributions (type III), using (5) and setting $y={\left[1+\exp(-x)\right]}^{-1}$, we obtain the result. For the generalized logistic distributions (type IV), using (5) and setting $y={\left[1+\exp(-x)\right]}^{-1}$, we obtain the result. For the generalized Pareto distributions [28], using the fact

∫0∞(x+β)−δlog(x+α)dx=ddγ∫0∞(x+β)−δ(x+α)γdxγ=0=ddγα1+γβ−δδ−γ−1F121,δ;δ−γ;1−αβγ=0,

we obtain the result. For the Gompertz distributions [29], using known expressions for expectations and the moment-generating function of a Gompertz random variable (see [29]), we obtain the result. For the half-Cauchy distributions, using the expansion (3) and known expressions for the expectations of a half-Cauchy random variable, we obtain the result. For the half-logistic distributions, using the expansion (2) and known expressions for the moment-generating function of a half-logistic random variable, we obtain the result. For the half-normal distributions, using known expressions for expectations of a half normal random variable, we obtain the result. For the half-Student’s *t*-distributions, using the expansion (3) and known expressions for expectations of a half-Student’s *t*-random variable, we obtain the result. For harmonic distributions, using known expressions for expectations of a harmonic random variable, we obtain the result. For the inverse chi-square distributions, using (4) and known expressions for expectations of an inverse chi-square random variable, we obtain the result. For the inverse exponential distributions, using known expressions for expectations of an inverse exponential random variable, we obtain the result. For the inverse gamma distributions, using (4) and known expressions for expectations of an inverse gamma random variable, we obtain the result. For the inverse Gaussian distributions [30], using known expressions for expectations of an inverse Gaussian random variable (see [30]), we obtain the result. For the inverse Nakagami distributions, using (4) and known expressions for expectations of an inverse Nakagami random variable, we obtain the result. For the inverse Rayleigh distributions, using known expressions for expectations of an inverse Rayleigh random variable, we obtain the result. For the *J*-shaped distributions [31], using

$$\begin{array}{c} \int_{0}^{1}\log xf\left(x\right)dx={\left.\frac{d}{d\alpha }\int_{0}^{1}x^{\alpha }f\left(x\right)dx\right|}_{\alpha =0}, \end{array}$$

$$\begin{array}{c} \int_{0}^{1}\log\left(2-x\right)f\left(x\right)dx={\left.\frac{d}{d\alpha }\int_{0}^{1}{\left(2-x\right)}^{\alpha }f\left(x\right)dx\right|}_{\alpha =0} \end{array}$$

and the definition of the incomplete beta function, we obtain the result. For the Kumaraswamy distributions [32], using the expansions given in (3) and (4) and known expressions for expectations of a Kumaraswamy random variable, we obtain the result. For the Laplace distributions, using known expressions for expectations of the absolute value of a Laplace random variable, we obtain the result. For Libby and Novick [33]’s beta distributions, using the facts

∫01xa−1(1−x)b−1logx(1+cx)a+bdx=ddγ∫01xa+γ−1(1−x)b−1(1+cx)a+bdxγ=0=ddγB(a+γ,b)F12a+γ,a+b;a+b+γ;−cγ=0,

∫01xa−1(1−x)b−1log(1−x)(1+cx)a+bdx=ddγ∫01xa−1(1−x)b+γ−1(1+cx)a+bdxγ=0=ddγB(a,b+γ)F12a,a+b;a+b+γ;−cγ=0

and

∫01xa−1(1−x)b−1log(1+cx)(1+cx)a+bdx=ddγ∫01xa−1(1−x)b−1(1+cx)a+b−γdxγ=0=ddγB(a,b)F12a,a+b−γ;a+b;−cγ=0,

we obtain the result. For the Lindley distributions [34], using known expressions for expectations of a Lindley random variable, we obtain the result. For the logit normal distributions, setting $y=\log\frac{x}{1-x}$ and integrating, we obtain the result. For the Lomax distributions [35], using the expansion (3) and known expressions for expectations of a Lomax random variable (see [35]), we obtain the result. For the Nakagami distributions [36], using (4) and known expressions for expectations of a Nakagami random variable (see [36]), we obtain the result. For the omega distributions [37], using the expansions given in (3) and (4) and known expressions for expectations of an omega random variable, we obtain the result. For Pareto type I distributions [38], straightforward calculations prove the result. For power function distributions of type I, straightforward calculations prove the result. For power function distributions of type II, straightforward calculations prove the result. For the *q*-exponential distributions [39], using the expansion given in (3) and known expressions for expectations of a *q*-exponential random variable (see [39]), we obtain the result. For scaled inverse chi-square distributions, using (4) and known expressions for expectations of a scaled inverse chi-square random variable, we obtain the result. For Schulz [40]’s distributions, using (4) and known expressions for expectations of a Schulz [40] random variable, we obtain the result. For the Student’s *t*-distributions, using the expansion given in (3) and known expressions for expectations of a Student’s *t*-random variable, we obtain the result. For the transmuted exponential distributions [42], using the expansion given in (2) and known expressions for the moment-generating function of a transmuted exponential random variable (see [42]), we obtain the result. For the truncated normal distributions, using known expressions for expectations of a truncated normal random variable, we obtain the result.

## 6. Numerical Check

**Figure 1:**
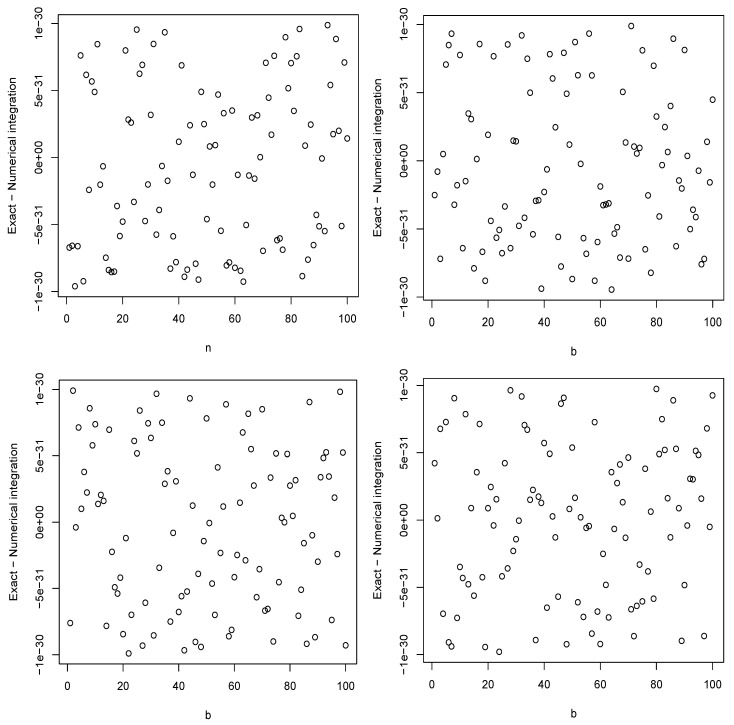
The differences between the values of (1) obtained by numerical integration and using the derived expressions for the chi distribution with m=1 and n=1,2,…,100 (**top left**); the exponential distribution with a=1 and b=1,2,…,100 (**top right**); the generalized logistic distribution of type I with a=1 and b=1,2,…,100 (**bottom left**); the power function distribution of type I with a=1 and b=1,2,…,100 (**bottom right**).

**Figure 2:**
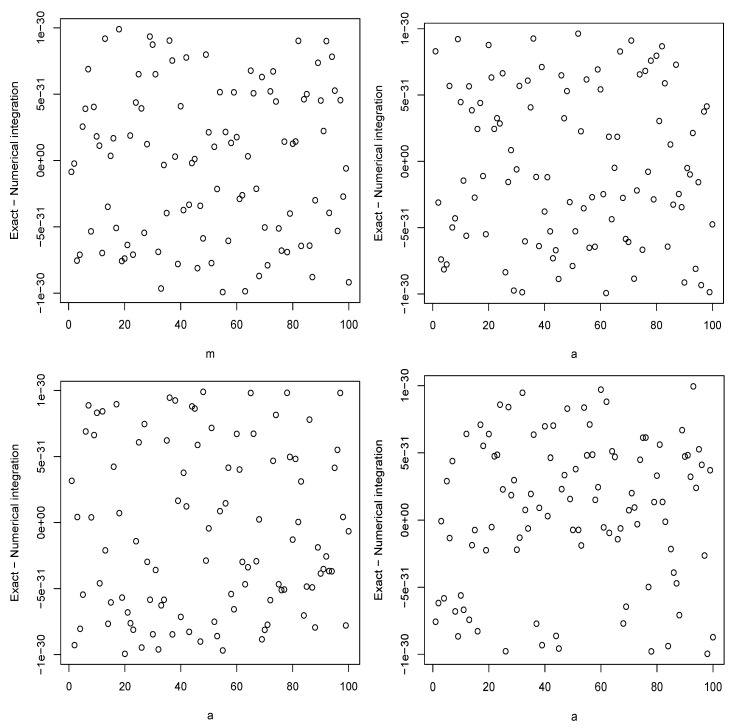
The differences between the values of (1) obtained by numerical integration and using the derived expressions for the chi distribution with n=1 and m=1,2,…,100 (**top left**); the exponential distribution with b=1 and a=1,2,…,100 (**top right**); the generalized logistic distribution of type I with b=1 and a=1,2,…,100 (**bottom left**); the power function distribution of type I with b=1 and a=1,2,…,100 (**bottom right**).

In this section, we check the correctness of the expressions given in Section 2 numerically. We compared values of (1) obtained by numerical integration and using the derived expressions. The comparisons are shown in Figure 1 and Figure 2 for the chi distribution, the exponential distribution, the generalized logistic distribution of type I and the power function distribution of type I.

The figures confirm the correctness of the expressions. Checks performed for the remaining sections not shown here also confirmed the correctness of all the expressions.

## 7. Conclusions

In this paper, we have presented precise mathematical formulations for the KLD pertaining to sixty-one continuous univariate distributions. We have rigorously verified the accuracy of these expressions to ensure their reliability. The extensive nature of our findings allows for their application across a diverse range of fields, including but not limited to electrical and electronic engineering, artificial intelligence, information systems, theory and methods in computer science, telecommunications, automation control systems, photographic technology, acoustics, software engineering, computational biology, biomedical engineering, operations research, management science, hardware architecture, remote sensing, neurosciences, optics, medical imaging, robotics, mechanical engineering, cybernetics, mechanics, industrial engineering, energy fuels, astronomy astrophysics, civil engineering, multidisciplinary geosciences, analytical chemistry, economics, physics fluids, chemical engineering, biology, transportation technology, medical informatics, aerospace engineering, environmental sciences, molecular biology, materials science, geochemistry, geophysics, mathematical psychology, genetics, heredity, linguistics, ecology, thermodynamics, health care sciences, atmospheric sciences, biophysics, legal medicine, fisheries, psychology, rehabilitation, ergonomics, forestry, zoology and behavioral sciences.

Looking ahead, we aim to extend our research by deriving exact expressions for the KLD in the context of discrete univariate distributions, which will further enhance the applicability of our work in various domains. This future endeavor will contribute to a more comprehensive understanding of the KLD across different types of data distributions.

## Figures and Tables

**Table 1:** Some known expressions for the KLD.

Distribution	$f_{1}\left(x\right)$ and $f_{2}\left(x\right)$	KLD	Due to
Confluent hypergeometric beta [10]	xa1−1(1−x)b1−1exp−c1xBa1,b1F11a1;a1+b1;−c1 and xa2−1(1−x)b2−1exp−c2xBa2,b2F11a2;a2+b2;−c2 for 0<x<1, $a_{1}>0$, $b_{1}>0$, $-\infty <c_{1}<\infty$, $a_{2}>0$, $b_{2}>0$ and $-\infty <c_{2}<\infty$	logBa2,b2F11a2;a2+b2;−c2Ba1,b1F11a1;a1+b1;−c1+a1−a2Γ′a1Γa1+ a1−a2∂∂αF11α+a1;α+a1+b1;−c1α=0F11a1;a1+b1;−c1−a1−a2Γ′a1+b1Γa1+b1+b1−b2Γ′b1Γb1+b1−b2∂∂αF11a1;α+a1+b1;−c1α=0F11a1;a1+b1;−c1−b1−b2Γ′a1+b1Γa1+b1+a1c2−c1a1+b1F111+a1;1+a1+b1;−c1F11a1;a1+b1;−c1	[14]
Exponential	aexp(−ax) and bexp(−bx) for x>0, a>0 and b>0	$\log\frac{a}{b}+\frac{b}{a}-1$	[15]
Gamma	$\frac{b^{a}x^{a-1}\exp\left(-bx\right)}{\Gamma (a)}$ and $\frac{d^{c}x^{c-1}\exp\left(-dx\right)}{\Gamma (c)}$ for x>0, a>0, b>0, c>0 and d>0	$a\log b-c\log d+\log\frac{\Gamma (c)}{\Gamma (a)}+\left(d-b\right)\frac{a}{b}+\left(a-c\right)\left[\psi (a)-\log b\right]$	[16]
Gauss hypergeometric beta [17]	xa1−1(1−x)b1−11+d1xc1Ba1,b1F12c1,a1;a1+b1;−d1 and xa2−1(1−x)b2−11+d2xc2Ba2,b2F12c2,a2;a2+b2;−d2 for 0<x<1, $a_{1}>0$, $b_{1}>0$, $-\infty <c_{1}<\infty$, $d_{1}>-1$, $a_{2}>0$, $b_{2}>0$, $-\infty <c_{2}<\infty$ and $d_{2}>-1$	logBa2,b2F12c2,a2;a2+b2;−d2Ba1,b1F12c1,a1;a1+b1;−d1+a1−a2Γ′a1Γa1+a1−a2∂∂αF12c1,α+a1;α+a1+b1;−d1α=0F12c1,a1;a1+b1;−d1−a1−a2Γ′a1+b1Γa1+b1+b1−b2Γ′b1Γb1+b1−b2∂∂αF12c1,a1;α+a1+b1;−d1α=0F12c1,a1;a1+b1;−d1−b1−b2Γ′a1+b1Γa1+b1+c2∂∂αF1a1,−α,c1;a1+b1;−d2,−d1α=0F12c1,a1;a1+b1;−d1−c1∂∂αF12c1−α,a1;a1+b1;−d1α=0F12c1,a1;a1+b1;−d1	[18]
Generalized gamma [11]	$\frac{a^{c}b}{\Gamma \left(\frac{c}{b}\right)}x^{c-1}\exp\left[-{\left(ax\right)}^{b}\right]$ and $\frac{d^{f}e}{\Gamma \left(\frac{f}{e}\right)}x^{f-1}\exp\left[-{\left(dx\right)}^{e}\right]$ for x>0, a>0, b>0, c>0, d>0, e>0 and f>0	$\log\left[\frac{a^{c}b\Gamma \left(\frac{f}{e}\right)}{d^{f}e\Gamma \left(\frac{c}{b}\right)}\right]+\frac{c-f}{\Gamma \left(\frac{c}{b}\right)}{\left.\frac{\partial }{\partial \alpha }\left[a^{-\alpha }\Gamma \left(\frac{c+\alpha }{b}\right)\right]\right|}_{\alpha =0}-\frac{c}{b}+\frac{d^{e}\Gamma \left(\frac{c+e}{b}\right)}{a^{e}\Gamma \left(\frac{c}{b}\right)}$	[19]
Gumbel [12]	$\frac{1}{a}\exp\left(-\frac{x-b}{a}\right)\exp\left[-\exp\left(-\frac{x-b}{a}\right)\right]$ and $\frac{1}{c}\exp\left(-\frac{x-d}{c}\right)\exp\left[-\exp\left(-\frac{x-d}{c}\right)\right]$ for −∞<x<∞, a>0, −∞<b<∞, c>0 and −∞<d<∞	$\log\frac{c}{a}+\left(\frac{1}{c}-\frac{1}{a}\right)\left(b+a\gamma \right)+\frac{b}{a}-\frac{d}{c}-1+\Gamma \left(1+\frac{a}{c}\right)\exp\left(\frac{d-b}{c}\right)$, where γ denotes Euler’s constant	[20]
Normal	$\frac{1}{a}\phi \left(\frac{x-b}{a}\right)$ and $\frac{1}{c}\phi \left(\frac{x-d}{c}\right)$ for −∞<x<∞, a>0, −∞<b<∞, c>0 and −∞<d<∞	$\log\frac{c}{a}+\frac{d^{2}}{2c^{2}}-\frac{b^{2}}{2a^{2}}+\frac{1}{2}\left(\frac{1}{c^{2}}-\frac{1}{a^{2}}\right)\left(a^{2}+b^{2}\right)+\left(\frac{b}{a^{2}}-\frac{d}{c^{2}}\right)b$	[21]
Rayleigh	$2b^{2}x\exp\left[-{\left(bx\right)}^{2}\right]$ and $2d^{2}x\exp\left[-{\left(dx\right)}^{2}\right]$ for x>0, b>0 and d>0	$2\log\left(\frac{b}{d}\right)+\frac{d^{2}}{b^{2}}-1$	[19]
Weibull [13]	$ab^{a}x^{a-1}\exp\left[-{\left(bx\right)}^{a}\right]$ and $cd^{c}x^{c-1}\exp\left[-{\left(dx\right)}^{c}\right]$ for x>0, a>0, b>0, c>0 and d>0	$\log\left(\frac{ab^{a}}{cd^{c}}\right)+\left(a-c\right){\left.\frac{\partial }{\partial \alpha }\left[\frac{1}{b^{\alpha }}\Gamma \left(1+\frac{\alpha }{a}\right)\right]\right|}_{\alpha =0}+\frac{d^{c}}{b^{a}}\Gamma \left(1+\frac{c}{a}\right)-1$	[19]

**Table 2:** New expressions for the KLD.

Distribution	$f_{1}\left(x\right)$ and $f_{2}\left(x\right)$	KLD
Generalized uniform	$\frac{\left(c+1\right)x^{c}}{b^{c+1}-a^{c+1}}$ and $\frac{\left(d+1\right)x^{d}}{b^{d+1}-a^{d+1}}$ for b>a>0, c>0, d>0 and a<x<b	$\log\left[\frac{c+1}{d+1}\frac{b^{d+1}-a^{d+1}}{b^{c+1}-a^{c+1}}\right]+\frac{\left(c+1)(c-d\right)}{b^{c+1}-a^{c+1}}\frac{\partial }{\partial c}\frac{b^{c+1}-a^{c+1}}{c+1}$
Asymmetric Laplace [22]	$\frac{ab}{a^{2}+1}\left\{\begin{array}{cc} \exp\left[\frac{b}{a}\left(x-c\right)\right], & ifx<c,\\ \exp\left[-ab(x-c)\right], & ifx\geq c, \end{array}\right.$ and $\frac{de}{d^{2}+1}\left\{\begin{array}{cc} \exp\left[\frac{e}{d}\left(x-c\right)\right], & ifx<c,\\ \exp\left[-de(x-c)\right], & ifx\geq c \end{array}\right.$ for a>0, b>0, −∞<c<∞, d>0, e>0 and −∞<x<∞	$\log\left(\frac{ab}{de}\frac{d^{2}+1}{a^{2}+1}\right)+\frac{a^{2}c}{a^{2}+1}\left(\frac{e}{d}-\frac{b}{a}\right)+\frac{c(ab-de)}{a^{2}+1}+\frac{a^{2}\left(cb-a\right)}{\left(a^{2}+1\right)b}\left(\frac{b}{a}-\frac{e}{d}\right)+\;\frac{de-ab}{a^{2}+1}\left(c+\frac{1}{ab}\right)$
Beta	$\frac{x^{a-1}{\left(1-x\right)}^{b-1}}{B(a,b)}$ and $\frac{x^{c-1}{\left(1-x\right)}^{d-1}}{B(c,d)}$ for a>0, b>0, c>0, d>0 and 0<x<1	$\log\left[\frac{B(c,d)}{B(a,b)}\right]+\left(a-c\right)\left[\psi (a)-\psi (a+b)\right]+\left(b-d\right)\left[\psi (b)-\psi (a+b)\right]$
Beta exponential [23]	$\frac{c}{B(a,b)}\exp\left(-bcx\right){\left[1-\exp(-cx)\right]}^{a-1}$ and $\frac{f}{B(d,e)}\exp\left(-efx\right){\left[1-\exp(-fx)\right]}^{d-1}$ for x>0, a>0, b>0, c>0, d>0, e>0 and f>0	$\log\left[\frac{cB(d,e)}{fB(a,b)}\right]+\left(\frac{ef}{c}-b\right)\left[\psi (a+b)-\psi (b)\right]-\left(a-1\right)\sum_{k=1}^{\infty }\frac{B(b+k,a)}{kB(a,b)}+\left(d-1\right)\sum_{k=1}^{\infty }\frac{B\left(b+\frac{kf}{c},a\;\right)}{kB\left(a,b\right)}$
Beta prime	$\frac{x^{a-1}{\left(1+x\right)}^{-a-b}}{B(a,b)}$ and $\frac{x^{c-1}{\left(1+x\right)}^{-c-d}}{B(c,d)}$ for a>0, b>0, c>0, d>0 and x>0	$\log\left[\frac{B(c,d)}{B(a,b)}\right]+\left(a-c\right)\left[\psi (a)-\psi (a+b)\right]+\left(b-d\right)\left[\psi (b)-\psi (a+b)\right]$
Burr [24]	$a^{b}bcx^{b-1}{\left[1+{\left(ax\right)}^{b}\right]}^{-c-1}$ and $d^{e}efx^{e-1}{\left[1+{\left(dx\right)}^{e}\right]}^{-f-1}$ for x>0, a>0, b>0, c>0, d>0, e>0 and f>0	$\log\left[\frac{a^{b}bc}{d^{e}ef}\right]+c\left(b-e\right){\left.\frac{\partial }{\partial \alpha }\left[a^{-\alpha }B\left(1+\frac{\alpha }{b},c-\frac{\alpha }{b}\right)\right]\right|}_{\alpha =0}-c\left(c+1\right)\sum_{k=1}^{\infty }\frac{{\left(-1\right)}^{k-1}}{k}B\left(1+k,c-k\right)+c\left(f+1\right)\sum_{k=1}^{\infty }\frac{{\left(-1\right)}^{k-1}d^{ke}}{ka^{ke}}B\left(1+\frac{ke}{b},c-\frac{ke}{b}\right)$
Cauchy	$\frac{1}{\pi \sigma }{\left(1+\frac{x^{2}}{\sigma^{2}}\right)}^{-1}$ and $\frac{1}{\pi r}{\left(1+\frac{x^{2}}{r^{2}}\right)}^{-1}$ for σ>0, r>0 and −∞<x<∞	$\log\frac{r}{\sigma }+\frac{r}{\sigma }\sum_{s=0}^{\infty }\left[\frac{{\left(\frac{1}{2}\right)}_{s}}{s!}{\left(1-\frac{r^{2}}{\sigma^{2}}\right)}^{s}\psi \left(s+1\right)\right]-\psi \left(1\right)$
Chi	$\frac{x^{m-1}\exp\left(-\frac{x^{2}}{2}\right)}{2^{\frac{m}{2}-1}\Gamma \left(\frac{m}{2}\right)}$ and $\frac{x^{n-1}\exp\left(-\frac{x^{2}}{2}\right)}{2^{\frac{n}{2}-1}\Gamma \left(\frac{n}{2}\right)}$ for m>0, n>0 and x>0	$\log\left[\frac{2^{\frac{n}{2}}\Gamma \left(\frac{n}{2}\right)}{2^{\frac{m}{2}}\Gamma \left(\frac{m}{2}\right)}\right]+\left(\frac{m}{2}-\frac{n}{2}\right)\left[\psi \left(\frac{m}{2}\right)-\log\left(\frac{1}{2}\right)\right]$
Chi-square	$\frac{x^{\frac{m}{2}-1}\exp\left(-\frac{x}{2}\right)}{2^{\frac{m}{2}}\Gamma \left(\frac{m}{2}\right)}$ and $\frac{x^{\frac{n}{2}-1}\exp\left(-\frac{x}{2}\right)}{2^{\frac{n}{2}}\Gamma \left(\frac{n}{2}\right)}$ for m>0, n>0 and x>0	$\log\left[\frac{2^{\frac{n}{2}}\Gamma \left(\frac{n}{2}\right)}{2^{\frac{m}{2}}\Gamma \left(\frac{m}{2}\right)}\right]+\left(\frac{m}{2}-\frac{n}{2}\right)\left[\psi \left(\frac{m}{2}\right)-\log\left(\frac{1}{2}\right)\right]$
Continuous Bernoulli	$C\left(a\right)a^{x}{\left(1-a\right)}^{1-x}$ and $C\left(b\right)b^{x}{\left(1-b\right)}^{1-x}$ for 0<a<1, 0<b<1 and 0<x<1, where $C\left(a\right)=\frac{2\;\mathrm{arctanh}(1-2a)}{1-2a}$ and $C\left(b\right)=\frac{2\;\mathrm{arctanh}(1-2b)}{1-2b}$	$\log\left[\frac{C(a)}{C(b)}\frac{1-a}{1-b}\right]+\left[\frac{a}{2a-1}+\frac{1}{2\;\mathrm{arctanh}(1-2a)}\right]\log\left[\frac{(1-b)a}{(1-a)b}\right]$
Exponentiated exponential [25]	$ab\exp\left(-bx\right){\left[1-\exp(-bx)\right]}^{a-1}$ and $cd\exp\left(-dx\right){\left[1-\exp(-dx)\right]}^{b-1}$ for x>0, a>0, b>0, c>0 and d>0	$\log\left(\frac{ab}{cd}\right)+\left(\frac{d}{b}-1\right)\left[\psi (a+1)-\psi (1)\right]-\left(a-1\right)\Gamma \left(a+1\right)\sum_{k=1}^{\infty }\frac{\Gamma (1+k)}{k\Gamma (1+k+a)}+\;\left(b-1\right)\Gamma \left(a+1\right)\sum_{k=1}^{\infty }\frac{\Gamma \left(1+\frac{kd}{b}\right)}{k\Gamma \left(1+\frac{kd}{b}+a\right)}$
*F*	$\frac{{\left(\frac{m}{n}\right)}^{\frac{m}{2}}x^{\frac{m}{2}-1}}{B\left(\frac{m}{2},\frac{n}{2}\right){\left(1+\frac{m}{n}x\right)}^{\frac{m+n}{2}}}$ and $\frac{{\left(\frac{k}{p}\right)}^{\frac{k}{2}}x^{\frac{k}{2}-1}}{B\left(\frac{k}{2},\frac{p}{2}\right){\left(1+\frac{k}{p}x\right)}^{\frac{k+p}{2}}}$ for m>0, n>0, k>0, p>0 and x>0	$\log\left[\frac{{\left(\frac{m}{n}\right)}^{\frac{m}{2}}B\left(\frac{k}{2},\frac{p}{2}\right)}{{\left(\frac{k}{p}\right)}^{\frac{k}{2}}B\left(\frac{m}{2},\frac{n}{2}\right)}\right]+\left(\frac{m-k}{2}\right)\left[\log\left(\frac{n}{m}\right)+\psi \left(\frac{m}{2}\right)\right]+\left(\frac{n-p}{2}\right)\psi \left(\frac{n}{2}\right)+\;\left(\frac{k+p}{2}\right){\left(\frac{mp}{nk}\right)}^{\frac{m}{2}}\sum_{s=0}^{\infty }\left[\frac{{\left(\frac{m}{2}\right)}_{s}}{s!}{\left(1-\frac{mp}{nk}\right)}^{s}\psi \left(s+\frac{m+n}{2}\right)\right]-\left(\frac{m+n}{2}\right)\psi \left(\frac{m+n}{2}\right)$
Fréchet [26]	$ab^{-a}x^{-a-1}\exp\left[-{\left(bx\right)}^{-a}\right]$ and $cd^{-c}x^{-c-1}\exp\left[-{\left(dx\right)}^{-c}\right]$ for x>0, a>0, b>0, c>0 and d>0	$\log\left(\frac{ad^{c}}{cb^{a}}\right)+\left(c-a\right){\left.\frac{\partial }{\partial \alpha }\left[\frac{1}{b^{\alpha }}\Gamma \left(1-\frac{\alpha }{a}\right)\right]\right|}_{\alpha =0}+\frac{b^{c}}{d^{c}}\Gamma \left(1-\frac{c}{a}\right)-1$
Gamma-Gompertz	$ab^{c}c\exp\left(ax\right){\left[b-1+\exp(ax)\right]}^{-c-1}$ and $de^{f}f\exp\left(dx\right){\left[e-1+\exp(dx)\right]}^{-f-1}$ for a>0, b>0, c>0, d>0, e>0, f>0 and x>0	logacbcdfef+dfac−1F12c,1;c+1;b−1b−(c+1)∑k=1∞(−1)k−1k(b−1)kbccc−kF12c+1,c−k;c−k+1;1−b+(f+1)∑k=1∞(−1)k−1k(e−1)kbcacac−kdF12c+1,c−kda;c−kda+1;1−b
Generalized beta of the first kind [27]	$\frac{ax^{ab-1}{\left(1-x^{a}\right)}^{c-1}}{B(b,c)}$ and $\frac{dx^{de-1}{\left(1-x^{d}\right)}^{f-1}}{B(e,f)}$ for a>0, b>0, c>0, d>0, e>0, f>0 and 0<x<1	$\log\left[\frac{aB(e,f)}{dB(b,c)}\right]+\left(ab-de\right){\left.\frac{\partial }{\partial \alpha }\frac{B\left(b+\frac{\alpha }{a},c\right)}{B(b,c)}\right|}_{\alpha =0}-\left(c-1\right)\sum_{k=1}^{\infty }\frac{1}{k}\frac{B\left(b+k,c\right)}{B(b,c)}+\left(f-1\right)\sum_{k=1}^{\infty }\frac{1}{k}\frac{B\left(b+\frac{kd}{a},c\right)}{B(b,c)}$
Generalized beta of the second kind [27]	$\frac{ax^{ac-1}}{b^{ac}B\left(c,d\right){\left[1+{\left(\frac{x}{b}\right)}^{a}\right]}^{c+d}}$ and $\frac{ex^{eg-1}}{f^{eg}B\left(g,h\right){\left[1+{\left(\frac{x}{f}\right)}^{e}\right]}^{g+h}}$ for a>0, b>0, c>0, d>0, e>0, f>0, g>0, h>0 and x>0	$\log\left[\frac{af^{eg}B\left(g,h\right)}{eb^{ac}B\left(c,d\right)}\right]+\left(ac-eg\right){\left.\frac{\partial }{\partial \alpha }\frac{b^{\alpha }B\left(c+\frac{\alpha }{a},d-\frac{\alpha }{a}\right)}{B(c,d)}\right|}_{\alpha =0}+\;\left(g+h\right)\sum_{k=1}^{\infty }\frac{{\left(-1\right)}^{k-1}}{k}\frac{b^{ek}B\left(c+\frac{ek}{a},d-\frac{ek}{a}\right)}{f^{ek}B\left(c,d\right)}-\left(c+d\right)\sum_{k=1}^{\infty }\frac{{\left(-1\right)}^{k-1}}{k}\frac{B\left(c+k,d-k\right)}{B(c,d)}$
Generalized inverse Gaussian	$\frac{1}{2K_{p}\left(\sqrt{ab}\right)}{\left(\frac{a}{b}\right)}^{\frac{p}{2}}x^{p-1}\exp\left[-\frac{1}{2}\left(ax+\frac{b}{x}\right)\right]$ and $\frac{1}{2K_{q}\left(\sqrt{cd}\right)}{\left(\frac{c}{d}\right)}^{\frac{q}{2}}x^{q-1}\exp\left[-\frac{1}{2}\left(cx+\frac{d}{x}\right)\right]$ for x>0, a>0, b>0, c>0, d>0, −∞<p<∞ and −∞<q<∞	$\log\left[\frac{{\left(\frac{a}{b}\right)}^{\frac{p}{2}}K_{q}\left(\sqrt{cd}\right)}{{\left(\frac{c}{d}\right)}^{\frac{q}{2}}K_{p}\left(\sqrt{ab}\right)}\right]+\left(p-q\right)\left\{\frac{1}{2}\log\left(\frac{b}{a}\right)+\frac{\partial }{\partial p}\log\left[K_{p}\left(\sqrt{ab}\right)\right]\right\}+\frac{1}{2}\left(c-a\right){\left(\frac{b}{a}\right)}^{\frac{1}{2}}\frac{K_{p+1}\left(\sqrt{ab}\right)}{K_{p}\left(\sqrt{ab}\right)}+\frac{1}{2}\left(d-b\right)\left[{\left(\frac{a}{b}\right)}^{\frac{1}{2}}\frac{K_{p+1}\left(\sqrt{ab}\right)}{K_{p}\left(\sqrt{ab}\right)}-\frac{2p}{b}\right]$
Generalized logistic (type I)	$\frac{a\exp(-x)}{{\left[1+\exp(-x)\right]}^{a+1}}$$\frac{b\exp(-x)}{{\left[1+\exp(-x)\right]}^{b+1}}$ for a>0, b>0 and −∞<x<∞	$\log\left(\frac{a}{b}\right)+\frac{b}{a}-1$
Generalized logistic (type II)	$\frac{a\exp(-ax)}{{\left[1+\exp(-x)\right]}^{a+1}}$ and $\frac{b\exp(-bx)}{{\left[1+\exp(-x)\right]}^{b+1}}$ for a>0, b>0 and −∞<x<∞	$\log\left(\frac{a}{b}\right)+\left(b-a\right)\left[\psi (a+1)-\psi (a)\right]$
Generalized logistic (type III)	$\frac{1}{B(a,a)}\frac{\exp(-ax)}{{\left[1+\exp(-x)\right]}^{2a}}$ and $\frac{1}{B(b,b)}\frac{\exp(-bx)}{{\left[1+\exp(-x)\right]}^{2b}}$ for a>0, b>0 and −∞<x<∞	$\log\left[\frac{B(b,b)}{B(a,a)}\right]+2\left(b-a\right)\left[\psi (2a)-\psi (a)\right]$
Generalized logistic (type IV)	$\frac{1}{B(a,b)}\frac{\exp(-bx)}{{\left[1+\exp(-x)\right]}^{a+b}}$ and $\frac{1}{B(c,d)}\frac{\exp(-dx)}{{\left[1+\exp(-x)\right]}^{c+d}}$ for a>0, b>0, c>0, d>0 and −∞<x<∞	$\log\left[\frac{B(c,d)}{B(a,b)}\right]+\left(a-c\right)\left[\psi (a)-\psi (a+b)\right]+\left(b-d\right)\left[\psi (b)-\psi (a+b)\right]$
Generalized Pareto [28]	$\frac{1}{b}{\left[1+a\frac{x-c}{b}\right]}^{-\frac{1}{a}-1}$ and $\frac{1}{e}{\left[1+d\frac{x-c}{e}\right]}^{-\frac{1}{d}-1}$ for a≥0, b>0, −∞<c<∞, d≥0, e>0 and x>0	logeb+1a+1d2a+1d+11a∂∂αbdacαB1d−α,α+1F121d−α;−α;1d+1;bd−aebdα=0
Gompertz [29]	$ab\exp\left[a+bx-a\exp(bx)\right]$ and $cd\exp\left[c+dx-c\exp(dx)\right]$ for x>0, a>0, b>0, c>0, d>0 and −∞<μ<∞	$\log\left(\frac{ab\;e^{a}}{cd\;e^{c}}\right)+\left(\frac{b-d}{b}\right)e^{a}\Gamma \left(0,a\right)+\frac{ce^{a}}{a^{\frac{d}{b}}}\Gamma \left(\frac{d}{b}+1,a\right)-a-1$
Half-Cauchy	$\frac{2}{\pi \sigma }{\left(1+\frac{x^{2}}{\sigma^{2}}\right)}^{-1}$ and $\frac{2}{\pi r}{\left(1+\frac{x^{2}}{r^{2}}\right)}^{-1}$ for σ>0, r>0 and x>0	$\log\frac{r}{\sigma }+\frac{r}{\sigma }\sum_{s=0}^{\infty }\left[\frac{{\left(\frac{1}{2}\right)}_{s}}{s!}{\left(1-\frac{r^{2}}{\sigma^{2}}\right)}^{s}\psi \left(s+1\right)\right]-\psi \left(1\right)$
Half-logistic	$\frac{2a\exp(-x)}{{\left[1+\exp(-x)\right]}^{a+1}}$ and $\frac{2b\exp(-x)}{{\left[1+\exp(-x)\right]}^{b+1}}$ for a>0, b>0 and x>0	$\log\left(\frac{a}{b}\right)+2a\left(b-a\right)\sum_{k=1}^{\infty }\frac{{\left(-1\right)}^{k-1}}{k}B_{\frac{1}{2}}\left(k+1,a\;-k\right)$
Half-normal	$\sqrt{2}a\exp\left[-{\left(ax\right)}^{2}\right]$ and $\sqrt{2}d\exp\left[-{\left(dx\right)}^{2}\right]$ for x>0, a>0 and d>0	$\log\frac{a}{d}-\frac{1}{2}+\frac{d^{2}}{2a^{2}}$
Half-Student’s *t*	$\frac{2}{\sigma \sqrt{n}\;B\left(\frac{1}{2},\frac{n}{2}\right)}{\left(1+\frac{x^{2}}{n\sigma^{2}}\right)}^{-\frac{n+1}{2}}$ and $\frac{2}{r\sqrt{p}\;B\left(\frac{1}{2},\frac{p}{2}\right)}{\left(1+\frac{x^{2}}{pr^{2}}\right)}^{-\frac{p+1}{2}}$ for n>0, p>0, σ>0, r>0 and x>0	$\log\left[\frac{r\sqrt{p}\;B\left(\frac{1}{2},\frac{p}{2}\right)}{\sigma \sqrt{n}\;B\left(\frac{1}{2},\frac{n}{2}\right)}\right]+\left(\frac{p+1}{2}\right){\left(\frac{pr^{2}}{n\sigma^{2}}\right)}^{\frac{1}{2}}\sum_{s=0}^{\infty }\left[\frac{{\left(\frac{1}{2}\right)}_{s}}{s!}{\left(1-\frac{pr^{2}}{n\sigma^{2}}\right)}^{s}\psi \left(s+\frac{n+1}{2}\right)\right]+\left(\frac{n-p}{2}\right)\psi \left(\frac{n}{2}\right)-\left(\frac{n+1}{2}\right)\psi \left(\frac{n+1}{2}\right)$
Harmonic	$\frac{1}{2xK_{0}\left(\alpha \right)}exp\left[-\frac{1}{2}\alpha \left(\frac{x}{m}+\frac{m}{x}\right)\right]$ and $\frac{1}{2xK_{0}\left(\beta \right)}exp\left[-\frac{1}{2}\beta \left(\frac{x}{n}+\frac{n}{x}\right)\right]$ for x>0, α>0, m>0, β>0 and n>0	$\log\left[\frac{K_{0}\left(\beta \right)}{K_{0}\left(\alpha \right)}\right]+\frac{1}{2}\beta \left(\frac{m}{n}+\frac{n}{m}\right)\frac{K_{1}\left(\alpha \right)}{K_{0}\left(\alpha \right)}-\alpha \frac{K_{1}\left(\alpha \right)}{K_{0}\left(\alpha \right)}$
Inverse chi-square	$\frac{x^{-\frac{m}{2}-1}\exp\left(-\frac{1}{2x}\right)}{2^{\frac{m}{2}}\Gamma \left(\frac{m}{2}\right)}$ and $\frac{x^{-\frac{n}{2}-1}\exp\left(-\frac{1}{2x}\right)}{2^{\frac{n}{2}}\Gamma \left(\frac{n}{2}\right)}$ for m>0, n>0 and x>0	$\log\left[\frac{2^{\frac{n}{2}}\Gamma \left(\frac{n}{2}\right)}{2^{\frac{m}{2}}\Gamma \left(\frac{m}{2}\right)}\right]+\left(\frac{m}{2}-\frac{n}{2}\right)\left[\psi \left(\frac{m}{2}\right)-\log\left(\frac{1}{2}\right)\right]$
Inverse exponential	$\frac{a}{x^{2}}\exp\left(-\frac{a}{x}\right)$ and $\frac{b}{x^{2}}\exp\left(-\frac{b}{x}\right)$ for x>0, a>0 and b>0	$\log a-\log b+\frac{b}{a}-1$
Inverse gamma	$\frac{b^{a}x^{-a-1}\exp\left(-\frac{b}{x}\right)}{\Gamma (a)}$ and $\frac{d^{c}x^{-c-1}\exp\left(-\frac{d}{x}\right)}{\Gamma (c)}$ for x>0, a>0, b>0, c>0 and d>0	$a\log b-c\log d+\log\frac{\Gamma (c)}{\Gamma (a)}+\left(d-b\right)\frac{a}{b}+\left(c-a\right)\left[\log b-\psi (a)\right]$
Inverse Gaussian [30]	$\sqrt{\frac{a}{2\pi x^{3}}}\exp\left[-\frac{a{\left(x-b\right)}^{2}}{2b^{2}x}\right]$ and $\sqrt{\frac{c}{2\pi x^{3}}}\exp\left[-\frac{c{\left(x-d\right)}^{2}}{2d^{2}x}\right]$ for x>0, a>0, b>0, c>0 and d>0	$\frac{1}{2}\log\frac{a}{c}+\frac{b}{2}\left(\frac{c}{d^{2}}-\frac{a}{b^{2}}\right)+\frac{a}{b}-\frac{c}{d}+\frac{c-a}{2}\left(\frac{1}{a}+\frac{1}{b}\right)$
Inverse Nakagami	$\frac{2a^{2m}}{\Gamma \left(m\right)}x^{-2m-1}\exp\left[-{\left(\frac{a}{x}\right)}^{2}\right]$ and $\frac{2d^{2n}}{\Gamma \left(n\right)}x^{-2n-1}\exp\left[-{\left(\frac{d}{x}\right)}^{2}\right]$ for x>0, a>0, d>0, m>0 and n>0	$\log\left[\frac{a^{2m}\Gamma \left(n\right)}{d^{2n}\Gamma \left(m\right)}\right]+\frac{2(m-n)}{\Gamma \left(m\right)}{\left.\frac{\partial }{\partial \alpha }\left[a^{-\alpha }\Gamma \left(\frac{2m+\alpha }{2}\right)\right]\right|}_{\alpha =0}-m+\frac{d^{2}m}{a^{2}}$
Inverse Rayleigh	$\frac{2b^{2}}{x^{3}}\exp\left[-{\left(\frac{b}{x}\right)}^{2}\right]$ and $\frac{2d^{2}}{x^{3}}\exp\left[-{\left(\frac{d}{x}\right)}^{2}\right]$ for x>0, b>0 and d>0	$2\log\left(\frac{b}{d}\right)+\frac{d^{2}}{b^{2}}-1$
*J*-shaped [31]	$2ax^{a-1}\left(1-x\right){\left(2-x\right)}^{a-1}$ and $2bx^{b-1}\left(1-x\right){\left(2-x\right)}^{b-1}$ for 0<x<1, a>0 and b>0	$\log\frac{a}{b}+\left(a-b\right)a4^{a}{\left.\frac{\partial }{\partial \alpha }\left\{2^{\alpha }\left[B_{\frac{1}{2}}\left(\alpha +a,a\right)-2B_{\frac{1}{2}}\left(1+\alpha +a,a\right)\right]\right\}\right|}_{\alpha =0}+\;\left(a-b\right)a4^{a}{\left.\frac{\partial }{\partial \alpha }\left\{2^{\alpha }\left[B_{\frac{1}{2}}\left(a,\alpha +a\right)-2B_{\frac{1}{2}}\left(a+1,\alpha +a\right)\right]\right\}\right|}_{\alpha =0}$
Kumaraswamy [32]	$abx^{a-1}{\left(1-x^{a}\right)}^{b-1}$ and $cdx^{c-1}{\left(1-x^{c}\right)}^{d-1}$ for 0<x<1, a>0, b>0, c>0 and d>0	$\log\left(\frac{ab}{cd}\right)+\left(a-c\right)b{\left.\frac{\partial }{\partial \alpha }B\left(1+\frac{\alpha }{a},b\right)\right|}_{\alpha =0}-\left(b-1\right)b\sum_{k=1}^{\infty }\frac{1}{k}B\left(1+k,b\right)+\;\left(d-1\right)b\sum_{k=1}^{\infty }\frac{1}{k}B\left(1+\frac{ck}{a},b\right)$
Laplace	$\frac{1}{2a}\exp\left(-\frac{|x-b|}{a}\right)$ and $\frac{1}{2c}\exp\left(-\frac{|x-d|}{c}\right)$ for −∞<x<∞, a>0, −∞<b<∞, c>0 and −∞<b<∞	$\log\frac{c}{a}+\frac{a}{c}\exp\left(-\frac{|b-d|}{a}\right)+\frac{|b-d|}{c}-1$
Libby and Novick [33]’s beta	$\frac{c_{1}^{a_{1}}x^{a_{1}-1}{\left(1-x\right)}^{b_{1}-1}}{B\left(a_{1},b_{1}\right){\left[1-\left(1-c_{1}\right)x\right]}^{a_{1}+b_{1}}}$ and $\frac{c_{2}^{a_{2}}x^{a_{2}-1}{\left(1-x\right)}^{b_{2}-1}}{B\left(a_{2},b_{2}\right){\left[1-\left(1-c_{2}\right)x\right]}^{a_{2}+b_{2}}}$ for 0<x<1, $a_{1}>0$, $b_{1}>0$, $c_{1}>0$, $a_{2}>0$, $b_{2}>0$ and $c_{2}>0$	logc1a1Ba2,b2c2a2Ba1,b1+c1a1a1−a2Ba1,b1∂∂αBa1+α,b1F12α+a1;a1+b1;α+a1+b1;1−c1α=0+c1a1b1−b2Ba1,b1∂∂αBa1,b1+αF12a1;a1+b1;α+a1+b1;1−c1α=0−c1a1a1+b1∂∂αF12a1;a1+b1−α;a1+b1;1−c1α=0+c1a1a2+b2∂∂αF1a1,a1+b1,−α,a1+b1;1−c1,1−c2α=0
Lindley [34]	$\frac{a^{2}}{a+1}\left(1+x\right)\exp\left(-ax\right)$ and $\frac{b^{2}}{b+1}\left(1+x\right)\exp\left(-bx\right)$ for x>0, a>0 and b>0	$\log\left[\frac{a^{2}}{b^{2}}\frac{b+1}{a+1}\right]+\frac{\left(a+2)(b-a\right)}{a(a+1)}$
Logit normal	$\frac{1}{ax(1-x)}\phi \left(-\frac{\mathrm{logit}\;x-b}{a}\right)$ and $\frac{1}{cx(1-x)}\phi \left(-\frac{\mathrm{logit}\;x-d}{c}\right)$ for a>0, −∞<b<∞, c>0, −∞<d<∞ and 0<x<1, where logit $x=\log\frac{x}{1-x}$	$\log\frac{c}{a}+\frac{1}{2c^{2}}{\left(b-d\right)}^{2}+\frac{a^{2}}{2c^{2}}-\frac{1}{2}$
Lomax [35]	$\frac{a}{b}{\left(1+\frac{x}{b}\right)}^{-a-1}$ and $\frac{c}{d}{\left(1+\frac{x}{d}\right)}^{-c-1}$ for a>0, b>0, c>0, d>0 and x>0	$\log\left(\frac{ad}{bc}\right)-\left(c+1\right)\psi \left(a\right)+\frac{(c+1)d}{b}\sum_{s=0}^{\infty }\left\{\frac{{\left(1\right)}_{s}}{s!}{\left(1-\frac{d}{b}\right)}^{s}\psi \left(s+1+a\right)\right\}-1-\frac{1}{a}$
Nakagami [36]	$\frac{2a^{2m}}{\Gamma \left(m\right)}x^{2m-1}\exp\left[-{\left(ax\right)}^{2}\right]$ and $\frac{2d^{2n}}{\Gamma \left(n\right)}x^{2n-1}\exp\left[-{\left(dx\right)}^{2}\right]$ for x>0, a>0, d>0, m>0 and n>0	$\log\left[\frac{a^{2m}\Gamma \left(n\right)}{d^{2n}\Gamma \left(m\right)}\right]+\frac{2(m-n)}{\Gamma \left(m\right)}{\left.\frac{\partial }{\partial \alpha }\left[a^{-\alpha }\Gamma \left(\frac{2m+\alpha }{2}\right)\right]\right|}_{\alpha =0}-m+\frac{d^{2}m}{a^{2}}$
Pareto type I [38]	$aK^{a}x^{-a-1}$ and $bK^{b}x^{-b-1}$ for x>K, a>0 and b>0	$\log\frac{a}{b}+\left(a-b\right)\log K+a\left(b-a\right){\left.\frac{\partial }{\partial \alpha }\left(\frac{K^{\alpha }}{a-\alpha }\right)\right|}_{\alpha =0}$
Power function of type I	$ax^{a-1}$ and $bx^{b-1}$ for 0<x<1, a>0 and b>0	$\log\frac{a}{b}+\frac{b}{a}-1$
Power function of type II	$a{\left(1-x\right)}^{a-1}$ and $b{\left(1-x\right)}^{b-1}$ for 0<x<1, a>0 and b>0	$\log\frac{a}{b}+\frac{b}{a}-1$
*q*-exponential [39]	$\left(2-a\right)b{\left[1-(1-a)bx\right]}^{\frac{1}{1-a}}$ and $\left(2-c\right)d{\left[1-(1-c)dx\right]}^{\frac{1}{1-c}}$ for a≥1, b>0, c≥1, d>0 and x>0	log(2−a)b(2−c)d−2−a(1−a)2∑k=1∞1k∑i=0kki(−1)ii+1+11−a+2−a1−c∑k=1∞(1−c)kdkk(1−a)k+1bk∑i=0kki(−1)ii+1+11−a
Scaled inverse chi-square	$\frac{{\left(\frac{a^{2}m}{2}\right)}^{\frac{m}{2}}}{\Gamma \left(\frac{m}{2}\right)}\frac{1}{x^{1+\frac{m}{2}}}\exp\left(-\frac{ma^{2}}{2x}\right)$ and $\frac{{\left(\frac{b^{2}n}{2}\right)}^{\frac{n}{2}}}{\Gamma \left(\frac{n}{2}\right)}\frac{1}{x^{1+\frac{n}{2}}}\exp\left(-\frac{nb^{2}}{2x}\right)$ for a>0, b>0, m>0, n>0 and x>0	$\log\left[\frac{{\left(\frac{a^{2}m}{2}\right)}^{\frac{m}{2}}\Gamma \left(\frac{n}{2}\right)}{{\left(\frac{b^{2}n}{2}\right)}^{\frac{n}{2}}\Gamma \left(\frac{m}{2}\right)}\right]+\left(\frac{n-m}{2}\right)\left[\log\left(a^{2}m\right)-\psi \left(\frac{m}{2}\right)\right]+\frac{nb^{2}-ma^{2}}{2a^{2}}$
Schulz [40]’s	$\frac{a^{a}x^{a-1}\exp\left(-ax\right)}{\Gamma (a)}$ and $\frac{b^{b}x^{b-1}\exp\left(-bx\right)}{\Gamma (b)}$ for x>0, a>0 and b>0	$a\log a-b\log b+\log\frac{\Gamma (b)}{\Gamma (a)}+b-a+\left(a-b\right)\left[\psi (a)-\log a\right]$
Student’s *t* [41]	$\frac{1}{\sigma \sqrt{n}\;B\left(\frac{1}{2},\frac{n}{2}\right)}{\left(1+\frac{x^{2}}{n\sigma^{2}}\right)}^{-\frac{n+1}{2}}$ and $\frac{1}{r\sqrt{p}\;B\left(\frac{1}{2},\frac{p}{2}\right)}{\left(1+\frac{x^{2}}{pr^{2}}\right)}^{-\frac{p+1}{2}}$ for n>0, p>0, σ>0, r>0 and −∞<x<∞	$\log\left[\frac{r\sqrt{p}\;B\left(\frac{1}{2},\frac{p}{2}\right)}{\sigma \sqrt{n}\;B\left(\frac{1}{2},\frac{n}{2}\right)}\right]+\left(\frac{p+1}{2}\right){\left(\frac{pr^{2}}{n\sigma^{2}}\right)}^{\frac{1}{2}}\sum_{s=0}^{\infty }\left[\frac{{\left(\frac{1}{2}\right)}_{s}}{s!}{\left(1-\frac{pr^{2}}{n\sigma^{2}}\right)}^{s}\psi \left(s+\frac{n+1}{2}\right)\right]+\;\left(\frac{n-p}{2}\right)\psi \left(\frac{n}{2}\right)-\left(\frac{n+1}{2}\right)\psi \left(\frac{n+1}{2}\right)$
Transmuted exponential [42]	$b\left[1-a+2a\exp(-bx)\right]\exp\left(-bx\right)$ and $d\left[1-c+2c\exp(-dx)\right]\exp\left(-dx\right)$ for a>0, b>0, c>0, d>0 and x>0	$\begin{array}{c} \log\left[\frac{(1-a)b}{(1-c)d}\right]-\left(\frac{d}{b}-1\right)\left(1-a+\frac{a}{c}\right)\\ +\sum_{k=1}^{\infty }\frac{{\left(-1\right)}^{k-1}}{k}{\left(\frac{2a}{1-a}\right)}^{k}\sum_{i=0}^{\infty }\frac{{\left(-kb\right)}^{i}\Gamma \left(i+1\right)}{i!b^{i}}\left(1-a+\frac{a}{2^{i}}\right)\\ -\sum_{k=1}^{\infty }\frac{{\left(-1\right)}^{k-1}}{k}{\left(\frac{2c}{1-c}\right)}^{k}\sum_{i=0}^{\infty }\frac{{\left(-kd\right)}^{i}\Gamma \left(i+1\right)}{i!b^{i}}\left(1-a+\frac{a}{2^{i}}\right) \end{array}$
Truncated normal	$\frac{\phi \left(\frac{x-b}{a}\right)}{a\left[\Phi \left(\frac{U-b}{a}\right)-\Phi \left(\frac{L-b}{a}\right)\right]}$ and $\frac{\phi \left(\frac{x-d}{c}\right)}{c\left[\Phi \left(\frac{U-d}{c}\right)-\Phi \left(\frac{L-d}{c}\right)\right]}$ for a>0, −∞<b<∞, c>0, −∞<d<∞ and L≤x≤U	$\begin{array}{c} \log\left\{\frac{c\left[\Phi \left(\frac{U-d}{c}\right)-\Phi \left(\frac{L-d}{c}\right)\right]}{a\left[\Phi \left(\frac{U-b}{a}\right)-\Phi \left(\frac{L-b}{a}\right)\right]}\right\}+\frac{d^{2}}{2c^{2}}-\frac{b^{2}}{2a^{2}}+\left(\frac{b}{a^{2}}-\frac{d}{c^{2}}\right)\\ \left[b-a\frac{\phi \left(\frac{U-b}{a}\right)-\phi \left(\frac{L-b}{a}\right)}{\Phi \left(\frac{U-b}{a}\right)-\Phi \left(\frac{L-b}{a}\right)}\right]\\ +\frac{1}{2}\left(\frac{1}{c^{2}}-\frac{1}{a^{2}}\right)\left[b^{2}-2a\frac{\phi \left(\frac{U-b}{a}\right)-\phi \left(\frac{L-b}{a}\right)}{\Phi \left(\frac{U-b}{a}\right)-\Phi \left(\frac{L-b}{a}\right)}\right]\\ +\frac{a}{2}\left(\frac{1}{c^{2}}-\frac{1}{a^{2}}\right)\frac{\left(U-b\right)\phi \left(\frac{U-b}{a}\right)-\left(L-b\right)\phi \left(\frac{L-b}{a}\right)}{\Phi \left(\frac{U-b}{a}\right)-\Phi \left(\frac{L-b}{a}\right)} \end{array}$
Omega [37]	$abc^{2b}\frac{x^{b-1}}{c^{2b}-x^{2b}}{\left(\frac{c^{b}+x^{b}}{c^{b}-x^{b}}\right)}^{-\frac{ac^{b}}{2}}$ and $dec^{2e}\frac{x^{e-1}}{c^{2e}-x^{2e}}{\left(\frac{c^{e}+x^{e}}{c^{e}-x^{e}}\right)}^{-\frac{dc^{e}}{2}}$ for a>0, b>0, d>0, e>0 and 0<x<c	logabc2(b−e)de−2(b+c)logc+a(b−e)cb∂∂α[cαBαb+1,acb2+1·F12acb2+1,αb+1;αb+acb2+1;−1]|α=0−acbacb2+1∑k=1∞(−1)k−1kBk+1,acb2+1·F12acb2+1,k+1;k+acb2+1;−1−acbacb2−1∑k=1∞1kBk+1,acb2+1·F12acb2+1,k+1;k+acb2+1;−1−acbdce2+1∑k=1∞(−1)k−1kBekb+1,acb2+1·F12acb2+1,ekb+1;ekb+acb2+1;−1−acbdce2−1∑k=1∞1kBekb+1,acb2+1·F12acb2+1,ekb+1;ekb+acb2+1;−1

## Data Availability

No new data were created or analyzed in this study. Data sharing is not applicable to this article.

## References

[1] Kullback S., Leibler R.A. (1951). On information and sufficiency. Ann. Math. Stat..

[2] Kullback S. (1959). Information Theory and Statistics.

[3] Liese F., Vajda I. (1987). Convex Statistical Distances.

[4] Vajda I. (1989). Theory of Statistical Inference and Information.

[5] Pardo L. (2006). Statistical Inference Based on Divergence Measures.

[6] Basu A., Shioya H., Park C. (2011). Statistical Inference: The Minimum Distance Approach.

[7] Nawa V., Nadarajah S. (2024). Exact expressions for Kullback-Leibler divergence for multivariate and matrix-variate distributions. Entropy.

[8] Prudnikov A.P., Brychkov Y.A., Marichev O.I. (1986). Integrals and Series.

[9] Gradshteyn I.S., Ryzhik I.M. (2000). Table of Integrals, Series, and Products.

[10] Gordy M.B. (1998). Computationally convenient distributional assumptions for common-value auctions. Comput. Econ..

[11] Stacy E.W. (1962). A generalization of the gamma distribution. Ann. Math. Stat..

[12] Gumbel E.J. (1941). The return period of flood flows. Ann. Math. Stat..

[13] Weibull W. (1951). A statistical distribution function of wide applicability. J. Appl. Mech..

[14] Nadarajah S., Kebe M. (2024). The confluent hypergeometric beta distribution. Mathematics.

[15] Choi B., Kim K., Song S.H. (2004). Goodness-of-fit test for exponentiality based on Kullback–Leibler information. Commun. Stat.-Simul. Comput..

[16] Mathiassen J.R., Skavhaug A., Bo K. (2022). Texture similarity measure using Kullback-Leibler divergence between gamma distributions. Lecture Notes in Computer Science.

[17] Armero C., Bayarri M.J. (1994). Prior assessments for prediction in queues. J. R. Stat. Soc. Ser. D Stat..

[18] Nadarajah S., Kebe M. (2024). The Gauss hypergeometric beta distribution. REVSTAT-Stat. J..

[19] Qin X., Zou H., Zhou S., Ji K. (2015). Region-based classification of SAR images using Kullback–Leibler distance between generalized gamma distributions,. IEEE Geosci. Remote Sens. Lett..

[20] Perez-Rodríguez P., Vaquera-Huerta H., Villasenor-Alva J.A. (2009). A goodness-of-fit test for the Gumbel distribution based on Kullback–Leibler information. Commun. Stat.—Theory Methods.

[21] Arizono I. (1989). and Ohta, H. A test for normality based on Kullback—Leibler information. Am. Stat..

[22] Kozubowski T.J., Podgorski K. (2000). A multivariate and asymmetric generalization of Laplace distribution. Comput. Stat..

[23] Nadarajah S., Kotz S. (2006). The beta exponential distribution. Reliab. Eng. Syst. Saf..

[24] Burr I.W. (1942). Cumulative frequency functions. Ann. Math. Stat..

[25] Gupta R.D., Kundu D. (2001). Exponentiated exponential family: An alternative to gamma and Weibull distributions. Biom. J..

[26] Fréchet M. (1927). Sur la loi de probabilite de l’ecart maximum. Ann. Soc. Polon. Math..

[27] McDonald J.B., Xu Y.J. (1995). A generalization of the beta distribution with applications. J. Econom..

[28] Pickands J. (1975). Statistical inference using extreme order statistics. Ann. Stat..

[29] Gompertz B. (1825). On the nature of the function expressive of the law of human mortality, and on a new mode of determining the value of life contingencies. Philos. Trans. R. Soc. Lond..

[30] Wald A. (1944). On cumulative sums of random variables. Ann. Math. Stat..

[31] Nadarajah S., Kotz S. (2003). Moments of some J-shaped distributions. J. Appl. Stat..

[32] Kumaraswamy P. (1980). A generalized probability density function for double-bounded random processes. J. Hydrol..

[33] Libby D.L., Novick M.R. (1982). Multivariate generalized beta-distributions with applications to utility assessment. J. Educ. Stat..

[34] Lindley D.V. (1958). Fiducial distributions and Bayes’ theorem. J. R. Stat. Soc. B.

[35] Lomax K.S. (1954). Business failures; Another example of the analysis of failure data. J. Am. Stat. Assoc..

[36] Nakagami N. The m-distribution a general formulation of intensity distribution of rapid fading. In *Statistical Methods in Radio Wave Propagation: Proceedings of a Symposium*; 1960; pp. 3–36. https://www.sciencedirect.com/science/article/abs/pii/B9780080093062500054.

[37] Dombi J., Jonas T., Toth E.Z., Arva G. (2019). The omega probability distribution and its applications in reliability theory. Qual. Reliab. Eng. Int..

[38] Pareto V. (1895). La legge della domanda. G. Degli Econ..

[39] Tsallis C. (2009). Nonadditive entropy and nonextensive statistical mechanics-an overview after 20 years. Braz. J. Phys..

[40] Schulz G.V. (1939). Uber die kinetik der kettenpolymerisationen. V. Z. Phys. Chem..

[41] Gosset W.S. (1908). The probable error of a mean. Biometrika.

[42] Shaw W.T., Buckley I.R.C. (2009). The alchemy of probability distributions: Beyond Gram-Charlier expansions, and a skew-kurtotic-normal distribution from a rank transmutation map. arXiv.

